# A nomogram incorporating treatment data for predicting overall survival in gastroenteropancreatic neuroendocrine tumors: a population-based cohort study

**DOI:** 10.1097/JS9.0000000000001080

**Published:** 2024-01-19

**Authors:** Zenghong Wu, Guochen Shang, Kun Zhang, Weijun Wang, Mengke Fan, Rong Lin

**Affiliations:** Division of Gastroenterology, Union Hospital, Tongji Medical College, Huazhong University of Science and Technology, Wuhan, China

**Keywords:** Decision curve analysis, gastroenteropancreatic neuroendocrine tumours, nomogram, overall survival

## Abstract

**Background:**

Over the last few decades, the annual global incidence of gastroenteropancreatic neuroendocrine tumours (GEP-NETs) has steadily increased. Because of the complex and inconsistent treatment of GEP-NETs, the prognosis of patients with GEP-NETs is still difficult to assess. The study aimed to construct and validate the nomograms included treatment data for prediction overall survival (OS) in GEP-NETs patients.

**Methods:**

GEP-NETs patients determined from the Surveillance, Epidemiology, and End Results (SEER)-13 registry database (1992–2018) and with additional treatment data from the SEER-18 registry database (1975–2016). In order to select independent prognostic factors that contribute significantly to patient survival and can be included in the nomogram, multivariate Cox regression analysis was performed using the minimum value of Akaike information criterion (AIC) and we analyzed the relationship of variables with OS by calculating hazard ratios (HRs) and 95% CIs. In addition, we also comprehensively compared the nomogram using to predict OS with the current 7th American Joint Committee on Cancer (AJCC) staging system.

**Results:**

From 2004 to 2015, a total of 42 662 patients at diagnosis years with GEP-NETs were determined from the SEER database. The results indicated that the increasing incidence of GEP-NETs per year and the highest incidence is in patients aged 50–54. After removing cases lacking adequate clinicopathologic characteristics, the remaining eligible patients (*n*=7564) were randomly divided into training (3782 patients) and testing sets (3782 patients). In the univariate analysis, sex, age, race, tumour location, SEER historic stage, pathology type, TNM, stage, surgery, radiation, chemotherapy, and CS tumour size were found to be significantly related to OS. Ultimately, the key factors for predicting OS were determined, involving sex, age, race, tumour location, SEER historic stage, M, N, grade, surgery, radiation, and chemotherapy. For internal validation, the C-index of the nomogram used to estimate OS in the training set was 0.816 (0.804–0.828). For external validation, the concordance index (C-index) of the nomogram used to predict OS was 0.822 (0.812–0.832). In the training and testing sets, our nomogram produced minimum AIC values and C-index of OS compared with AJCC stage. Decision curve analysis (DCA) indicated that the nomogram was better than the AJCC staging system because more clinical net benefits were obtained within a wider threshold probability range.

**Conclusion:**

A nomogram combined treatment data may be better discrimination in predicting overall survival than AJCC staging system. The authors highly recommend to use their nomogram to evaluate individual risks based on different clinical features of GEP-NETs, which can improve the diagnosis and treatment outcomes of GEP-NETs patients and improve their quality of life.

## Background

Highlights

- The study aimed to construct and validate the nomograms included treatment data for prediction overall survival in gastroenteropancreatic neuroendocrine tumours (GEP-NETs) patients.
- The results indicated that the increasing incidence of GEP-NETs per year and the highest incidence is in patients aged 50–54.
- Decision curve analysis indicated that the nomogram was better than the American Joint Committee on Cancer staging system.

The gastroenteropancreatic neuroendocrine tumours (GEP-NETs) are class of heterogeneous rare tumours, origin from the diffuse neuroendocrine system of the pancreas and gastrointestinal tract. Over the last few decades, the annual global incidence of GEP-NETs has steadily increased. The projected prevalence of NETs in the US population in 2014 was 171 321^1^. The increased incidence of GEP-NETs may be associated with the rise of the exposure to risk factors and with the advanced endoscopic examination in clinical. GEP-NETs were related to many risk factors. The achlorhydria is a redisposing condition of type I gastric NETs in atrophic gastritis^2^. Feola *et al*.^3^ highlights the role of family history based on the three-centre retrospective case-control study and confirms type 2 diabetes mellitus (T2DM) and obesity as independent risk factors for GEP-NETs. Alcohol consumption and cigarette smoking are potential risk factors for selected anatomical sites^4^. In addition, the worsening of clinicopathological features in GEP-NETs is related to the higher presence of metabolic syndrome^5^. The diagnosis of GEP-NETs relies on clinical presentation, pathology, as well as conventional or functional imaging for accurate assessment^6^. It is recommended that patients display symptoms of carcinoid syndrome undergo plasma 5-hydroxyindoleacetic acid (5-HIAA) testing^7^. Markers of neuroendocrine differentiation can be identified through immunohistochemistry, which including synaptophysin, chromogranin A (CgA), neuron-specific enolase (NSE), and cluster of differentiation 56 (CD56), these markers can used to identify the primary site of metastatic tumours^8^. Surgical intervention is typically the first choice for localized, non-metastatic disease, followed by systemic chemotherapy. Patients with advanced GEP-NETs often experience limited treatment options due to disease progression or intolerance to chemotherapy regimens. However, combination immunotherapy and new potential compounds may offer clinical benefits^9–18^. Moreover, GEP-NETs may be more aggressive and associated with a poorer prognosis^19^. For advanced patients at the time of initial diagnosis, the 5-year survival rates are 57% for well-differentiated grade 3 (G3) GEP-NETs and only 5.2% for small-cell tumours^20^. Thus, because of poor prognosis and inconsistent treatment of GEP-NETs, the prognosis of patients with GEP-NETs is still difficult to assess.

Tumour-node-metastasis (TNM) staging system, which defined by American Joint Commission on Cancer (AJCC), has been normally established formula for predicting patient’s prognosis of malignancies for a long time^21^. The using of AJCC staging system fails to assess the individualized survival involved in patients’ clinicopathologic characteristics, but just roughly classified patients into different groups. Nevertheless, other factors, such as age, sex, race, tumour site and size, and treatment strategy can also have an obvious impact on patients’ prognosis. Within the currently accessible prediction means, nomograms are deemed as the high precision and forecasting ability graphic and quantitative models for predicting the survival of patients with cancer^22^. Compared with the AJCC staging system, nomogram can estimate survival for individual patients more accurately by integrating important prognostic variables. Xu *et al*.^23^ constructed a nomogram with 5 prognostic parameters to quantify the risk of patients’ death with GEP-NETs, but did not explore the effect of treatment factors, such as surgery, chemotherapy and radiation. Krieg *et al.* ^24^ constructed a nomogram to estimate the prognosis of GEP-NETs patients after surgery and Wu *et al*.^25^ constructed a predictive model of GEP-NETs grading using preoperative laboratory and imaging parameters, while the relatively small sample size is the limitation for both works. Until now, no studies have combined substantial treatment factors with clinical factors to predict the prognosis of patients with GEP-NETs based on the nomogram. Thus, the novelty of this work to construct and validate the nomograms incorporating treatment data for overall survival (OS) prediction based on the Surveillance, Epidemiology, and End Results (SEER) GEP-NETs cohort study, and also to contrast the performance of our nomograms with that of the AJCC staging system.

## Materials and methods

### Study population

**Figure 1:**
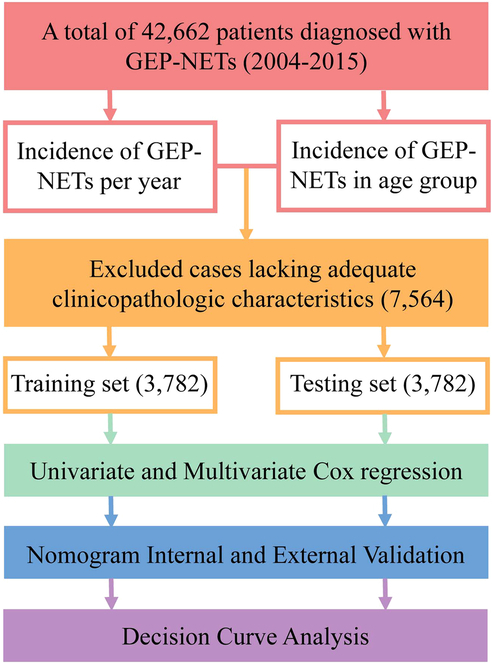
A flow chart of the study. GEP-NET, gastroenteropancreatic neuroendocrine tumour.

A flow chart of the study procedure was shown in Fig. 1. We firstly identified GEP-NETs patients from the SEER-13 registry database (1992–2018) and with additional treatment data from the SEER-18 registry database (1975–2016) using National Cancer Institute (NCI)’s SEER*Stat software (version 8.3.9). Histologic codes from the International Classification of Diseases for Oncology, Third Edition (ICD-O-3), and site codes to determine GEP-NETs patients reference Xu *et al.* ^23^s’ criterion. GEP-NETs were diagnosed as the merely primary malignancy based on histology. The TNM staging data were retrieved according to the following codes: Derived AJCC Stage Group, 6th ed (2004–2015), Derived AJCC T, 6th ed (2004–2015), Derived AJCC N, 6th ed (2004–2015), Derived AJCC M, 6th ed (2004–2015). Diagnostic information can only be obtained from patients with death certificates or autopsy reports, and patients who died within 1 month after the initial diagnosis were excluded. Our study does not require the approval of the institutional review committee because the SEER database is freely available for global researchers. The work has been reported on line with the STROCSS criteria^26^.

### Study variables

Variables used in SEER database, including age (from age 20–24 to 85+ years), sex (female, male), race (white, black, and others (American Indian/AK Native, Asian/Pacific Islander)), tumour size (reclassified as ≤20, 21–40, and ≥41), SEER stage, pathology, histologic grade, surgery, radiotherapy, chemotherapy, survival months, and vital status for individual patient, were extracted from the SEER database. SEER historic stage A (1973–2015) was used for GEP-NETs staging: localized, regional, unstaged, and distant. For treatment data, SEER offers therapy classification such as chemotherapy, surgery, and radiotherapy based on SEER-specific records. We choose surgery using the SEER code “Reason no cancer-direct surgery”, while radiotherapy and chemotherapy were determined as those using “radiation recode” and “chemotherapy recode” in SEER. As for tumour categories, the SEER categories scheme systematically classifies the subjects into four grades: grade 1 with high discrimination (G1), moderately differentiated (G2), poorly differentiated (G3), and undifferentiated or anaplastic (G4). The Site recode ICD-O-3/WHO 2008 information were used to filter by tumour location, involving appendix, ascending colon, caecum, descending colon, hepatic flexure, large intestine, pancreas, rectosigmoid junction, rectum, sigmoid colon, small intestine, splenic flexure, stomach, and transverse colon. Age was reclassified as younger than or equal to 30, 31–60, and older than or equal to 61. OS were defined as the primary endpoints of the study.

### Patient selection

We selected patients from the SEER database based on following criteria: confirmation of the tumour through histological examination, presence of a single primary tumour (either the first of at least 2 primaries), availability of complete staging information, and survival for more than 1 month. We identified all GEP-NETs cases by using histologic and site codes. Patients with unknown treatment intervention were not included in the study when constructing the nomogram. In the modified staging system, a new T category called T1b was introduced to include patients with tumours measuring 1–2 cm that did not invade the muscularis propria. Patients were excluded if their staging data were incomplete or the tumour originated from a site other than the duodenum. Patients with other secondary cancers were also excluded.

### Statistical analysis

After removing cases lacking adequate clinicopathologic characteristics, the remaining eligible patients (*n*=7564) were randomly divided into 1:1 training and testing sets using a computer-generated randomized, permuted-block scheme. Multivariate Cox regression models were applied to assess characteristics related to OS. Multivariate Cox regression analysis was performed using the minimum value of Akaike information criterion (AIC), the reverse stepwise process stop rule, and the independent prognostic factors that significantly contributed to patients’ survival were selected to construct the nomogram, as well as the nomogram of 3-year and 5-year OS. The verification of nomograms is mainly based on internal (training set) and external (testing set) discrimination and calibration measurements. Consistency index (C-index) mainly measures the difference between the predicted results and the actual results and is the main index to evaluate the discrimination ability of nomogram. The calibration curve was applied to compare the predicted survival rate and the actual survival rate according to the nomogram. In order to minimize the overfitting bias, the nomograms were subjected to 1000 bootstrap resamples in both validations.

Decision curve analysis (DCA) begins by assuming that the threshold probability, for which a patient would choose treatment, provides useful information on how the patient considers the risks associated with a false-positive and a false-negative prediction. This theoretical connection is then utilized to calculate the overall benefit of the model across various threshold probabilities. By graphing the net benefit against the threshold probability, we obtain the “decision curve”. DCA identifies the range of threshold probabilities wherein a model is beneficial, determines the extent of the benefit, and determines the most optimal model out of several options. DCA was used to explore the clinical practicability of nomogram in predicting survival, and the clinical value of nomogram was compared with AJCC staging system in training and testing cohort, respectively.

Descriptive statistics *t*-tests or χ2 tests were applied to compare patients’ basic clinical characteristics. Assumptions was conducted based on Shapiro–Wilk test, and the assumptions are fulfilled. Cox multivariable regression was performed to analysis the relationship of variables with OS by calculating hazard ratios (HRs) and 95% CIs. Before conducting Cox multivariable regression, collinearity diagnosis was performed on the variables, and the results showed that the tolerance values of the included variables were all less than 1 and the Variance Influence Factor values were all less than 10. Therefore, there is no multicollinearity among the included variables in the study, and the constructed model is relatively stable. The clinicopathologic characteristics on patient survival was analyzed applying the Kaplan–Meier curve and compared using the log-rank test. Cox multivariable regression analyses were conducted with SPSS, version 23 (IBM Corp) and OS analyses were conducted with GraphPad Prism 8 XML project. Nomogram and calibration curve was performed based on the “rms” R package and DCA analysis based on the “ggDCA” R package. For each analysis, the statistically significant setting was *P* less than 0.05.

## Results

### Patient characteristics

**Figure 2:**
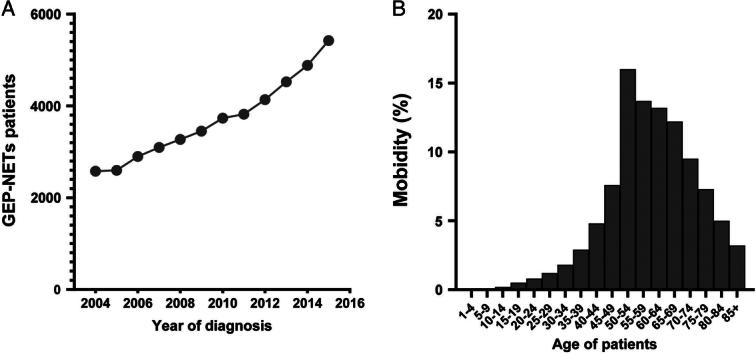
The incidence of gastroenteropancreatic neuroendocrine tumours (GEP-NETs) in Surveillance, Epidemiology, and End Result database and the results indicated that the increasing incidence of GEP-NETs per year and the highest incidence is in patients aged 50–54. (A) Year group; (B). Age group.

From 2004 to 2015, a total of 42 662 patients at diagnosis years with GEP-NETs were determined from the SEER database. The results indicated that the increasing incidence of GEP-NETs per year and the highest incidence was in patients aged 50–54 Fig. 2. After removing cases lacking adequate clinicopathologic characteristics, the remaining eligible patients (*n*=7564) were randomly divided into training (3782 patients) and testing sets (3782 patients). The demographic and clinicopathological characteristics in both training set and testing set by sex were summarized in Table S1, Supplemental Digital Content 1, http://links.lww.com/JS9/B741. Among the patients in training set, the majority of GEP-NETs patients were white in both female (78.6%) and male (81.7%), most male patients (35.6%) were at SEER regional stage, and most patients were at grade I categories. The most frequent tumour site was the pancreas and small intestine, training set showed a comparable trend with testing sets in both female and male patients. Among all the patients, the majority of patients experienced surgery and chemotherapy, while most of patients did not have undergone radiotherapy. Regarding as CS tumour size, greater than or equal to 41 mm was the most common.

### Prognostic nomograms incorporating treatment data

**Figure 3:**
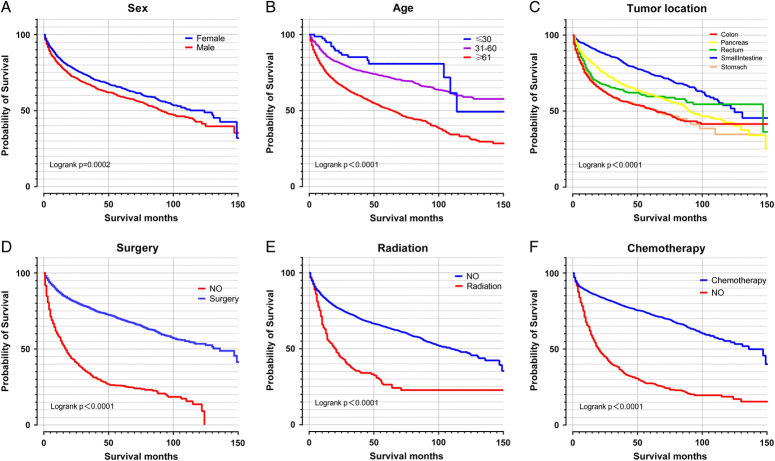
The clinicopathologic characteristics on patients’ overall survival in gastroenteropancreatic neuroendocrine tumours and the results suggested that sex, age, tumour location, surgery, and chemotherapy was significance impact patients’ prognostic. (A) Sex; (B) Age; (C) Tumour location; (D) Surgery; (E). Radiation; (F). Chemotherapy.

**Figure 4:**
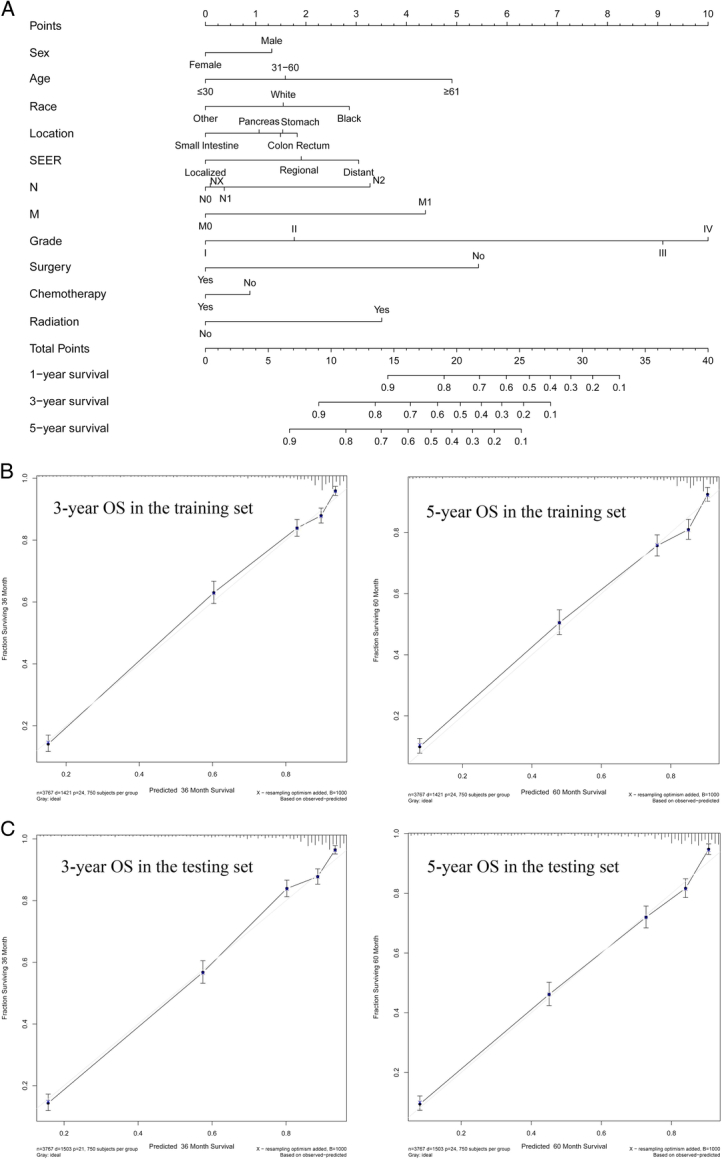
Nomogram and the calibration curves. (A) Nomogram for predicting 3-year and 5-year overall survival for gastroenteropancreatic neuroendocrine tumours; (B) The calibration curves of overall survival nomograms in the training set; (C) The calibration curves of overall survival nomograms in the testing set. OS, overall survival; SEER, Surveillance, Epidemiology, and End Result.

We next explored some variables on patients OS, and the results suggested that sex, age, tumour location, surgery, and chemotherapy had significance impact on patients’ prognostic. Male, older patients, colon, and absence of surgery or chemotherapy was related to poor prognostic. Interestingly, we found that radiotherapy was associated with the poor prognostic (may be due to the limited sample size) Fig. 3. In the univariate analysis, sex, age, race, tumour location, SEER historic stage, pathology type, TNM, stage, surgery, radiation, chemotherapy, and CS tumour size were found to be significantly related to OS. In the following multivariate Cox regression, all these significant factors were firstly incorporated into Cox regression model. In order to select independent prognostic factors that contributed significantly to patient survival and that can be included in the nomogram, we take the minimum value of AIC for variable selection. Ultimately, the key factors for predicting OS were determined, involving sex, age, race, tumour location, SEER historic stage, M, N, grade, surgery, radiation, and chemotherapy Table S2, Supplemental Digital Content 2, http://links.lww.com/JS9/B742. These factors were also included in the nomogram used to predict 3-year and 5-year OS Fig. 4A. Meanwhile, the univariate and multivariate Cox regression in texting set was shown in Table S3, Supplemental Digital Content 3, http://links.lww.com/JS9/B743.

### Nomogram internal and external validation

For internal validation, the C-index of the nomogram used to estimate OS in the training set was 0.816 (0.804–0.828). For external validation, the C-index of the nomogram used to predict OS was 0.822 (0.812–0.832). The results of C-index suggested that our nomogram was suitable for GEP-NETs patients. The calibration curves of 3-year OS nomograms and 5-year OS (Fig. 4B) in the training indicated the best consistency among our nomogram prediction and actual survival. The calibration curves of 3-year OS nomograms and 5-year OS (Fig. 4C) in the testing set was also explored. In addition, we also comprehensively compared the nomogram used to predict OS with the current 7th AJCC staging system. In the training and testing set, our nomogram produced minimum AIC values and C-index of OS compared with AJCC stage Table S4, Supplemental Digital Content 4, http://links.lww.com/JS9/B744, with *P* values less than 0.001 among all groups. The results showed that our nomogram had robust and more accurate prediction ability than the traditional AJCC staging system, which is consistent with previous published literature^25–27^.

### Decision curve analysis of nomogram prediction models

**Figure 5:**
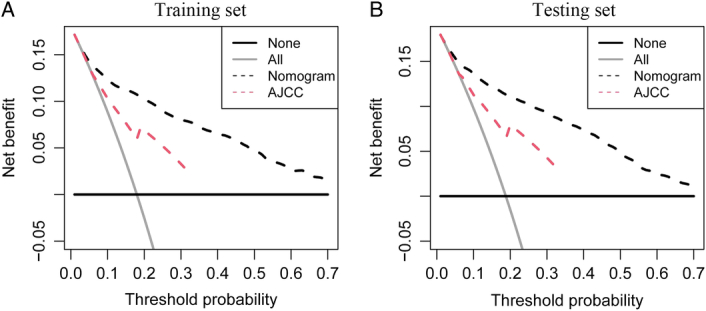
Decision curve analysis among the nomograms and American Joint Committee on Cancer (AJCC) staging system of overall survival for gastroenteropancreatic neuroendocrine tumours and our nomogram was better than the AJCC staging system both in training set and testing set. (A) Training set; (B). Testing set.

After solving the accuracy of the model, the training set was used to perform DCA to make the nomogram clinically practical, and it was extended to the testing set. Nomograms had high clinical potential in predicting OS since they had a wide and practical threshold probability range through 3 or 5 years of OS in both sets. Moreover, our nomogram was better than the AJCC staging system because more clinical net benefits were obtained within a wider threshold probability range using nomogram than using AJCC staging Fig. 5, which means that our nomogram can aid clinical decision-making and improve outcomes for our patients.

## Discussion

In this study, we used a large amount of data integrated in the SEER program to analyze the series of GEP-NETs cases reported up to 2015, and incorporated treatment data to construct a novel convenient and comprehensive nomogram for assessing the 3-year and 5-year OS of GEP-NETs patients. Our nomogram showed satisfactory discrimination performance and accuracy in both external and internal verification. Moreover, our nomogram achieved higher values regarding in both C-index and DCA assessment systems compared with AJCC TNM. Furthermore, through the nomogram, we could determine patients with various prognosis, so as to promote the follow-up plan and individualized treatment of this tumour with persistent increase incidence.

Nomogram integrates diverse parameters (such as treatment data) to evaluate the probability of survival and exhibited higher accuracy than the current AJCC staging system, so it has been recognized as an alternative and even a new staging system^28–30^. Previous studies have demonstrated the predictive ability of nomograms for GEP-NETs^23,31–33^, but it all failed to combined with therapy data, such as surgery, chemotherapy and radiation. Only a small part of GEP-NETs patients with metastatic tumours can receive surgical management at the time of diagnosis^34^. Chemotherapy is the majority advisable therapeutic choice for GEP-NETs patients. Because of higher proliferative index, these patients are sensitive to chemotherapy^35^. Platinum-based (carboplatin or cisplatin) chemotherapy is most widely used in GEP-NETs patients^36^. ^177^Lu-DOTATATE was approved by FDA in 2018 for therapy of GEP-NETs and constitutes a better advancement in the treatment of these diseases^37^. Study indicated that treatment with radiopeptides may temporarily result in radiation-induced hormone disturbances^38^. Multiple rounds of ^131^I-MIBG were related to prolonged survival^39^. In our study, we found that absence of surgery or chemotherapy was related to the poor prognostic and that radiotherapy was associated with the poor prognostic. There are limited RCT data on the overall activity and safety of radiotherapy in GEP-NETs. Moreover, due to the relatively insufficient radiotherapy sample size we included, it may affect the accuracy of the results. Study indicated that a large release of IL-1a from tumour cells after radiotherapy can cause oxidative DNA damage mediated cellular senescence, which leads to patients’ resistance to radiotherapy and causes disease progression^40^. Meanwhile, radiotherapy to the prostate did not improve overall survival for unselected patients with newly diagnosed metastatic prostate cancer^41^. Since SEER database lacks comprehensive information on radiotherapy, it may also be a source of bias. Therefore, we emphasized that radiation therapy should be administered to specific selected patients.

The nomogram also includes the primary tumour location which has not been included as variables in the JACC staging systems. Previous studies have demonstrated the predictive ability of nomograms for GEP-NETs located in the pancreas^42^, colon^43^, and small intestine^44^. Our study involved GEP-NETs originating from the rectum, colon, small intestine, stomach, and pancreas as the study characteristics and determined the primary tumour location as an independent prognostic factor. Studies suggested that patients with rectum GEP-NETs have best prognosis, whereas colon or pancreas GEP-NETs patients appear to have poorer outcomes^1,45^. Our results are consistent with these studies as we found that colon or pancreas patients shown poorer outcomes and small intestine shown better prognostic. Epidemiological inconsistencies have been reported recently between American and European countries versus Asian countries. The rectum and pancreas are the most common sites of onset in Asian populations, while the midgut and pancreas are the most common sites of onset in white Europeans and Americans. In our study, we found that both female and male patients appear to be more prone to developing NETs of the small intent and pancreas. Therefore, when formulating relevant policies, attention should be paid to this difference. This result reinforces the critical role of primary tumour location for GEP-NETs. The constructed nomograms for GEP-NETs patients made it possible to predict the prognosis individually. Our results indicated that tumour grade demonstrated higher HRs than other involved factors, which means that tumour grade was the most beneficial independent prognostic factor for GEP-NETs patients. Søreide *et al*.^46^ found that tumour grade, age, and primary surgical therapy were independent prognostic factors for OS in GEP-NETs patients. Moreover, higher tumour grade and pancreatic NETs recommended more frequent surveillance as grade factor predicted distant recurrence^47^. In order to select more independent prognostic factors that contributed significantly to patient survival, we took the minimum value of AIC for variable selection and determined 11 significant prognostic parameters (sex, age, race, tumour location, SEER historic stage, M, N, grade, surgery, radiation, and chemotherapy), which could provide simple and accurate prognostic predictions for patients with GEP-NETs. For example, a female White (1.56 points) 30-year-old patient with small intestine NETs, N2 (3.2 points), M1 (4.4 points), SEER localized with grade III (9 points), absence of surgery intervenes (5.4 points), absence of radiation and chemotherapy intervene (0.8 points) would have a 1-year survival rate of 58% (24.36 points) and a 3-year survival rate of 26% (24.36 points) according to our nomogram. In addition, our nomogram showed better discrimination in predicting OS than 7th AJCC staging system since our nomogram was based on a larger population in SEER database. More importantly, we used DCA to explore clinical beneficial of our nomogram, which showed that the nomogram had wider clinical applicability compared with current AJCC staging system. Particularly, since the nomogram development is based on SEER in contrast to the globally validated AJCC system, the cross-country applicability of the nomogram is limited to the population used to train the algorithm.

Our study also has several limitations. Firstly, nomogram was constructed using retrospective data from SEER database, which may introduce some inevitable deviations, such as treatment selection deviation and missing data. Secondly, due to the lack of Ki-67 and mitotic index in SEER database, which are keys for tumour classification, these factors are not considered in our tumour classification strategy. Several important prognostic factors, such as relapse free survival, microvascular invasion, vascular resection performed, R0/R1 resection, antitumor immune response, functional or non-functional GEP-NETs, genomic panels and peptide receptor radionuclide therapy were not taken into consideration in our study. In addition, the points assigned in the nomogram were based on various clinical and pathological factors that have been shown to be associated with prognosis in GEP tumours. These factors include tumour size, differentiation grade, lymph node involvement, metastasis, and other relevant characteristics. While the nomogram takes into account of these factors, it does not directly capture the specific survival outcomes of each tumour type separately. Therefore, it is essential to carry out large-scale research to explicate these factors in the future. The study identifies radiotherapy is related to poor prognosis without adequately considering the limited sample size of radiotherapy cases, potentially introducing bias. Finally, the nomogram needs further in-depth refine, and prospective validation with other independent patients was important. Incorporating advanced artificial intelligence techniques, such as radiomics and pathomics, can significantly enhance our understanding of disease characteristics, improve prognostic prediction, and facilitate personalized treatment approaches. The inclusion of deep learning-based predictive models in future studies can add valuable insights and improve the accuracy of prognostic predictions.

## Conclusion

A nomogram combined treatment data were constructed and validated that evaluated 3-year and 5-year overall survival based on the population-based data in GEP-NETs patients and showed better discrimination in predicting overall survival than AJCC staging system.

## Ethical approval

The data of our study were derived from SEER database. All procedures performed in studies involving human participants were in accordance with the ethical standards of the institutional and national research committee and with the 1964 Helsinki Declaration and its later amendments or comparable ethical standards. The SEER Program collects data from population-based cancer registries with anonymous information. The SEER is a publicly available database, thus no ethical approval is required.

## Source of funding

This study was supported by the National Key R&D Program of China (grant numbers 2023YFC2307000) and National Natural Science Foundation of China [grant numbers 82170571 and 81974068].

## Author contribution

Z.W.: methodology, formal analysis, writing—original draft. W.W.: methodology, formal analysis, writing—original draft. K.Z.: methodology, formal analysis, writing—original draft. G.S.: methodology, formal analysis, writing—original draft. M.F.: provided design improvement, administrative and material support, supervision. R.L.: provided design improvement, administrative and material support, supervision.

## Conflicts of interest disclosure

The authors have no potential conflicts of interest to disclose.

## Research registration unique identifying number (UIN)

Name of the registry: Research Registry Unique Identifying number or registration ID: Researchregistry8747 Hyperlink to your specific registration (must be publicly accessible and will be checked): https://www.researchregistry.com/browse-theregistry#home/registrationdetails/6409861cedff4d0029e67c68/.

## Guarantor

Dr. Wu, and Lin had full access to all of the data in the study and take responsibility for the integrity of the data and the accuracy of the data analysis.

## Data availability statement

All data used in this study can be freely accessed from the SEER program (https://seer.cancer.gov/).

## Additional contribution

The interpretation and reporting of these data are the sole responsibility of the authors. The authors acknowledge the efforts of the National Cancer Institute and the Surveillance, Epidemiology, and End Results (SEER) Program tumour registries in the creation of the SEER database.

## Originality of content

The authors verify that all information and materials in the manuscript are original.

## Supplementary Material

SUPPLEMENTARY MATERIAL
