# Supplementary material for: A nomogram incorporating treatment data for predicting overall survival in gastroenteropancreatic neuroendocrine tumors: a population-based cohort study
Source: Int J Surg. 2024 Jan 19;110(4):2178–86. doi: 10.1097/JS9.0000000000001080 (PMC11020034; doi:10.1097/JS9.0000000000001080)
Supplement: SUPPLEMENTARY MATERIAL [file js9-110-2178-s001.docx]

| **Characteristics** | **Training Set** |  |  | **Testing Set** |  |  |
| --- | --- | --- | --- | --- | --- | --- |
|  | **Male (N=1974)** | **Female (N=1808)** | ***P*-value** | **Male (N=1944)** | **Female (N=1838)** | ***P*-value** |
| **Age** |  |  |  |  |  |  |
| ≤30 | 51 (2.6%) | 59 (3.3%) | 0.219 | 27 (1.4%) | 51 (2.8%) | 0.00459 |
| 31-60 | 816 (41.3%) | 777 (43.0%) |  | 811 (41.7%) | 794 (43.2%) |  |
| ≥61 | 1107 (56.1%) | 972 (53.8%) |  | 1106 (56.9%) | 993 (54.0%) |  |
| **Race** |  |  |  |  |  |  |
| Black | 266 (13.5%) | 315 (17.4%) | 0.0023 | 211 (10.9%) | 238 (12.9%) | 0.0363 |
| Other | 95 (4.8%) | 72 (4.0%) |  | 175 (9.0%) | 190 (10.3%) |  |
| White | 1613 (81.7%) | 1421 (78.6%) |  | 1558 (80.1%) | 1410 (76.7%) |  |
| **Tumor location** |  |  |  |  |  |  |
| Colon | 408 (20.7%) | 436 (24.1%) | 0.0155 | 381 (19.6%) | 465 (25.3%) | <0.001 |
| Pancreas | 578 (29.3%) | 457 (25.3%) |  | 613 (31.5%) | 533 (29.0%) |  |
| Rectum | 213 (10.8%) | 213 (11.8%) |  | 233 (12.0%) | 185 (10.1%) |  |
| Small Intestine | 604 (30.6%) | 533 (29.5%) |  | 552 (28.4%) | 494 (26.9%) |  |
| Stomach | 171 (8.7%) | 169 (9.3%) |  | 165 (8.5%) | 161 (8.8%) |  |
| **SEER historic stage** |  |  |  |  |  |  |
| Distant | 702 (35.6%) | 613 (33.9%) | 0.0398 | 711 (36.6%) | 642 (34.9%) | 0.256 |
| Localized | 650 (32.9%) | 666 (36.8%) |  | 627 (32.3%) | 639 (34.8%) |  |
| Regional | 622 (31.5%) | 529 (29.3%) |  | 606 (31.2%) | 557 (30.3%) |  |
| **Stage** |  |  |  |  |  |  |
| I | 558 (28.3%) | 590 (32.6%) | 0.0241 | 534 (27.5%) | 552 (30.0%) | 0.131 |
| II | 306 (15.5%) | 269 (14.9%) |  | 348 (17.9%) | 294 (16.0%) |  |
| III | 469 (23.8%) | 382 (21.1%) |  | 393 (20.2%) | 392 (21.3%) |  |
| IV | 641 (32.5%) | 567 (31.4%) |  | 669 (34.4%) | 600 (32.6%) |  |
| **T** |  |  |  |  |  |  |
| T1 | 509 (25.8%) | 522 (28.9%) | 0.112 | 485 (24.9%) | 479 (26.1%) | 0.589 |
| T2 | 351 (17.8%) | 322 (17.8%) |  | 359 (18.5%) | 321 (17.5%) |  |
| T3 | 664 (33.6%) | 595 (32.9%) |  | 690 (35.5%) | 652 (35.5%) |  |
| T4 | 324 (16.4%) | 280 (15.5%) |  | 295 (15.2%) | 294 (16.0%) |  |
| TX | 126 (6.4%) | 89 (4.9%) |  | 115 (5.9%) | 92 (5.0%) |  |
| **N** |  |  |  |  |  |  |
| N0 | 963 (48.8%) | 945 (52.3%) | 0.0338 | 939 (48.3%) | 923 (50.2%) | 0.0173 |
| N1 | 793 (40.2%) | 644 (35.6%) |  | 785 (40.4%) | 686 (37.3%) |  |
| N2 | 154 (7.8%) | 160 (8.8%) |  | 137 (7.0%) | 167 (9.1%) |  |
| N3 | 0 (0%) | 0 (0%) |  | 0 (0%) | 1 (0.1%) |  |
| NX | 64 (3.2%) | 59 (3.3%) |  | 83 (4.3%) | 61 (3.3%) |  |
| **M** |  |  |  |  |  |  |
| M0 | 1334 (67.6%) | 1241 (68.6%) | 0.485 | 1276 (65.6%) | 1242 (67.6%) | 0.214 |
| M1 | 640 (32.4%) | 567 (31.4%) |  | 668 (34.4%) | 596 (32.4%) |  |
| **Grade** |  |  |  |  |  |  |
| Grade I | 1129 (57.2%) | 1092 (60.4%) | 0.0237 | 1048 (53.9%) | 1013 (55.1%) | 0.319 |
| Grade II | 304 (15.4%) | 296 (16.4%) |  | 358 (18.4%) | 338 (18.4%) |  |
| Grade III | 404 (20.5%) | 324 (17.9%) |  | 391 (20.1%) | 376 (20.5%) |  |
| Grade IV | 137 (6.9%) | 96 (5.3%) |  | 147 (7.6%) | 111 (6.0%) |  |
| **Surgery** |  |  |  |  |  |  |
| No | 367 (18.6%) | 277 (15.3%) | 0.00822 | 357 (18.4%) | 296 (16.1%) | 0.0706 |
| Yes | 1607 (81.4%) | 1531 (84.7%) |  | 1587 (81.6%) | 1542 (83.9%) |  |
| **Radiation** |  |  |  |  |  |  |
| No | 1860 (94.2%) | 1716 (94.9%) | 0.389 | 1818 (93.5%) | 1755 (95.5%) | 0.00841 |
| Yes | 114 (5.8%) | 92 (5.1%) |  | 126 (6.5%) | 83 (4.5%) |  |
| **Chemotherapy** |  |  |  |  |  |  |
| No | 1477 (74.8%) | 1407 (77.8%) | 0.0322 | 1476 (75.9%) | 1439 (78.3%) | 0.0886 |
| Yes | 497 (25.2%) | 401 (22.2%) |  | 468 (24.1%) | 399 (21.7%) |  |
| **CS tumor size (mm)** |  |  |  |  |  |  |
| 21-40 | 492 (24.9%) | 445 (24.6%) | <0.001 | 497 (25.6%) | 499 (27.1%) | 0.0516 |
| ≥41 | 809 (41.0%) | 637 (35.2%) |  | 804 (41.4%) | 689 (37.5%) |  |
| ≤20 | 673 (34.1%) | 726 (40.2%) |  | 643 (33.1%) | 650 (35.4%) |  |
| Data expressed as a number (percentage). | | | | | | |
