# Supplementary material for: A nomogram incorporating treatment data for predicting overall survival in gastroenteropancreatic neuroendocrine tumors: a population-based cohort study
Source: Int J Surg. 2024 Jan 19;110(4):2178–86. doi: 10.1097/JS9.0000000000001080 (PMC11020034; doi:10.1097/JS9.0000000000001080)
Supplement: SUPPLEMENTARY MATERIAL [file js9-110-2178-s002.docx]

| **Characteristics** | **Univariate** |  | **Multivariate (Full model)** |  | **Multivariate (AIC-based model)** |  |
| --- | --- | --- | --- | --- | --- | --- |
|  | **HR (95% CI)** | ***P*-value** | **HR (95% CI)** | ***P*-value** | **HR (95% CI)** | ***P*-value** |
| **Sex** |  |  |  |  |  |  |
| **Female** | Reference |  | Reference |  | Reference |  |
| **Male** | 1.222 (1.101-1.357) | ＜0.001 | 1.214 (1.091-1.349) | ＜0.001 | 1.238 (1.113-1.376) | ＜0.001 |
| **Age** |  |  |  |  |  |  |
| ≤30 | Reference |  | Reference |  | Reference |  |
| 31-60 | 2.267 (1.332-3.857) | 0.003 | 1.340 (0.786-2.284) | 0.283 | 1.306 (0.766-2.227) | 0.326 |
| ≥61 | 3.915 (2.310-6.637) | ＜0.001 | 2.339 (1.376-3.975) | 0.002 | 2.266 (1.333-3.852) | 0.003 |
| **Race** |  |  |  |  |  |  |
| Black | Reference |  | Reference |  | Reference |  |
| White | 1.099 (0.949-1.272) | 0.209 | 0.842 (0.725-0.979) | 0.025 | 0.824 (0.708-0.957) | 0.011 |
| Other | 0.793 (0.578-1.088) | 0.150 | 0.666 (0.484-0.916) | 0.013 | 0.638 (0.463-0.878) | 0.006 |
| **Tumor location** |  |  |  |  |  |  |
| Colon | Reference |  | Reference |  | Reference |  |
| Pancreas | 0.720 (0.627-0.827) | ＜0.001 | 0.883 (0.749-1.041) | 0.137 | 0.928 (0.783-1.099) | 0.387 |
| Rectum | 0.756 (0.630-0.906) | 0.003 | 1.033 (0.847-1.261) | 0.748 | 1.056 (0.865-1.290) | 0.592 |
| Small Intestine | 0.418 (0.359-0.486) | ＜0.001 | 0.740 (0.617-0.887) | 0.001 | 0.775 (0.642-0.937) | 0.008 |
| Stomach | 1.004 (0.836-1.206) | 0.962 | 0.936 (0.766-1.143) | 0.515 | 0.978 (0.798-1.200) | 0.835 |
| **SEER historic stage** |  |  |  |  |  |  |
| Distant | Reference |  | Reference |  | Reference |  |
| Localized | 0.177 (0.152-0.205) | ＜0.001 | 0.591 (0.411-0.850) | 0.005 | 0.595 (0.413-0.857) | 0.005 |
| Regional | 0.309 (0.273-0.351) | ＜0.001 | 0.847 (0.607-1.184) | 0.331 | 0.818 (0.585-1.142) | 0.238 |
| **Stage** |  |  | Not selected |  | Not selected |  |
| I | Reference |  |  |  |  |  |
| II | 1.826 (1.482-2.250) | ＜0.001 |  |  |  |  |
| III | 1.798 (1.485-2.176) | ＜0.001 |  |  |  |  |
| IV | 6.316 (5.382-7.411) | ＜0.001 |  |  |  |  |
| **T** |  |  | Not selected |  | Not selected |  |
| T1 | Reference |  |  |  |  |  |
| T2 | 1.374 (1.141-1.655) | ＜0.001 |  |  |  |  |
| T3 | 1.761 (1.507-2.507) | ＜0.001 |  |  |  |  |
| T4 | 3.354 (2.871-3.919) | ＜0.001 |  |  |  |  |
| **N** |  |  |  |  |  |  |
| N0 | Reference |  | Reference |  | Reference |  |
| N1 | 1.364 (1.212-1.534) | ＜0.001 | 1.023 (0.887-1.180) | 0.753 | 1.064 (0.922-1.228) | 0.395 |
| N2 | 3.631 (3.159-4.174) | ＜0.001 | 1.297 (1.098-1.531) | 0.002 | 1.719 (1.398-2.114) | ＜0.001 |
| **M** |  |  |  |  |  |  |
| M0 | Reference |  | Reference |  | Reference |  |
| M1 | 4.357 (3.918-4.844) | ＜0.001 | 2.018 (1.456-2.796) | ＜0.001 | 2.060 (1.487-2.854) | ＜0.001 |
| **Grade** |  |  |  |  |  |  |
| Grade I | Reference |  | Reference |  | Reference |  |
| Grade II | 1.580 (1.336-1.869) | ＜0.001 | 1.322 (1.116-1.567) | 0.001 | 1.344 (1.134-1.593) | ＜0.001 |
| Grade III | 6.931 (6.125-7.843) | ＜0.001 | 4.213 (3.646-4.868) | ＜0.001 | 4.543 (3.901-5.290) | ＜0.001 |
| Grade IV | 8.056 (6.788-9.562) | ＜0.001 | 4.965 (4.118-5.987) | ＜0.001 | 5.330 (4.398-6.459) | ＜0.001 |
| **Surgery** |  |  |  |  |  |  |
| No | Reference |  | Reference |  | Reference |  |
| Yes | 0.266 (0.238-0.297) | ＜0.001 | 0.469 (0.408-0.540) | ＜0.001 | 0.400 (0.344-0.465) | ＜0.001 |
| **Radiation** |  |  |  |  |  |  |
| No | Reference |  | Reference |  | Reference |  |
| Yes | 2.642 (2.220-3.144) | ＜0.001 | 1.673 (1.376-2.034) | ＜0.001 | 1.820 (1.488-2.227) | ＜0.001 |
| **Chemotherapy** |  |  | Not selected |  | Reference |  |
| No | Reference |  |  |  | Reference |  |
| Yes | 3.693 (3.322-4.106) | ＜0.001 |  |  | 0.856 (0.747-0.981) | 0.025 |
| **CS tumor size (mm)** |  |  | Not selected |  | Not selected |  |
| ≤20 | Reference |  |  |  |  |  |
| 21-40 | 1.840 (1.566-2.163) | ＜0.001 |  |  |  |  |
| ≥41 | 3.802 (3.313-4.363) | ＜0.001 |  |  |  |  |
|  |  |  |  |  |  |  |
