# Supplementary material for: A nomogram incorporating treatment data for predicting overall survival in gastroenteropancreatic neuroendocrine tumors: a population-based cohort study
Source: Int J Surg. 2024 Jan 19;110(4):2178–86. doi: 10.1097/JS9.0000000000001080 (PMC11020034; doi:10.1097/JS9.0000000000001080)
Supplement: SUPPLEMENTARY MATERIAL [file js9-110-2178-s003.docx]

| **Characteristics** | **Univariate** |  | **Multivariate (Full model)** |  | **Multivariate (AIC-based model)** |  |
| --- | --- | --- | --- | --- | --- | --- |
|  | **HR (95% CI)** | ***P*-value** | **HR (95% CI)** | ***P*-value** | **HR (95% CI)** | ***P*-value** |
| **Sex** |  |  |  |  |  |  |
| **Female** | Reference |  | Reference |  | Reference |  |
| **Male** | 1.196 (1.080-1.323) | 0.001 | 1.206 (1.088-1.337) | ＜0.001 | 1.217 (1.098-1.350) | ＜0.001 |
| **Age** |  |  |  |  |  |  |
| ≤30 | Reference |  | Reference |  | Reference |  |
| 31-60 | 1.431 (0.869-2.356) | 0.159 | 1.384 (0.840-2.283) | 0.202 | 1.325 (0.803-2.189) | 0.270 |
| ≥61 | 2.917 (1.780-4.780) | ＜0.001 | 2.567 (1.564-4.215) | ＜0.001 | 2.442 (1.485-4.016) | ＜0.001 |
| **Race** |  |  |  |  |  |  |
| Black | Reference |  | Not selected |  | Not selected |  |
| White | 1.072 (0.912-1.260) | 0.402 |  |  |  |  |
| Other | 0.912 (0.723-1.149) | 0.433 |  |  |  |  |
| **Tumor location** |  |  |  |  |  |  |
| Colon | Reference |  | Reference |  | Reference |  |
| Pancreas | 0.613 (0.537-0.699) | ＜0.001 | 0.817 (0.696-0.959) | 0.013 | 0.805 (0.685-0.947) | 0.009 |
| Rectum | 0.719 (0.602-0.858) | ＜0.001 | 0.869 (0.715-1.056) | 0.158 | 0.867 (0.714-1.053) | 0.149 |
| Small Intestine | 0.420 (0.362-0.488) | ＜0.001 | 0.721 (0.605-0.858) | ＜0.001 | 0.749 (0.625-0.898) | 0.002 |
| Stomach | 1.078 (0.904-1.284) | 0.403 | 1.056 (0.870-1.282) | 0.580 | 1.057 (0.872-1.282) | 0.571 |
| **SEER historic stage** |  |  | Not selected |  |  |  |
| Distant | Reference |  |  |  | Reference |  |
| Localized | 0.190 (0.164-0.219) | ＜0.001 |  | 0.005 | 0.750 (0.625-0.898) | 0.167 |
| Regional | 0.385 (0.342-0.433) | ＜0.001 |  | 0.331 | 1.103 (0.757-1.608) | 0.609 |
| **Stage** |  |  |  |  | Not selected |  |
| I | Reference |  | Reference |  |  |  |
| II | 2.110 (1.728-2.576) | ＜0.001 | 1.336 (1.075-1.661) | 0.009 |  |  |
| III | 2.283 (1.889-2.759) | ＜0.001 | 1.576 (1.234-2.014) | ＜0.001 |  |  |
| IV | 6.324 (5.374-7.442) | ＜0.001 | 3.404 (2.754-4.206) | ＜0.001 |  |  |
| **T** |  |  | Not selected |  | Not selected |  |
| T1 | Reference |  |  |  |  |  |
| T2 | 1.564 (1.294-1.891) | ＜0.001 |  |  |  |  |
| T3 | 2.238 (1.908-2.624) | ＜0.001 |  |  |  |  |
| T4 | 4.131 (3.508-4.866) | ＜0.001 |  |  |  |  |
| **N** |  |  |  |  |  |  |
| N0 | Reference |  | Reference |  | Reference |  |
| N1 | 1.438 (1.281-1.613) | ＜0.001 | 1.041 (0.903-1.200) | 0.577 | 1.053 (0.916-1.211) | 0.466 |
| N2 | 4.111 (3.591-4.705) | ＜0.001 | 1.378 (1.170-1.624) | ＜0.001 | 1.614 (1.326-1.964) | ＜0.001 |
| **M** |  |  |  |  |  |  |
| M0 | Reference |  | Not selected |  | Reference |  |
| M1 | 3.744 (3.381-4.147) | ＜0.001 |  |  | 2.542 (1.751-3.692) | ＜0.001 |
| **Grade** |  |  |  |  |  |  |
| Grade I | Reference |  | Reference |  | Reference |  |
| Grade II | 1.875 (1.610-2.183) | ＜0.001 | 1.480 (1.266-1.728) | ＜0.001 | 1.499 (1.283-1.752) | ＜0.001 |
| Grade III | 6.605 (5.835-7.477) | ＜0.001 | 4.025 (3.466-4.674) | ＜0.001 | 4.384 (3.750-5.124) | ＜0.001 |
| Grade IV | 8.249 (6.980-9.749) | ＜0.001 | 4.167 (3.451-5.031) | ＜0.001 | 4.512 (3.716-5.480) | ＜0.001 |
| **Surgery** |  |  |  |  |  |  |
| No | Reference |  | Reference |  | Reference |  |
| Yes | 0.308 (0.276-0.344) | ＜0.001 | 0.490 (0.423-0.567) | ＜0.001 | 0.439 (0.376-0.513) | ＜0.001 |
| **Radiation** |  |  |  |  |  |  |
| No | Reference |  | Reference |  | Reference |  |
| Yes | 2.594 (2.193-3.067) | ＜0.001 | 1.571 (1.296-1.903) | ＜0.001 | 1.685 (1.385-2.050) | ＜0.001 |
| **Chemotherapy** |  |  | Not selected |  | Reference |  |
| No | Reference |  |  |  | Reference |  |
| Yes | 3.460 (3.120-3.837) | ＜0.001 |  |  | 0.876 (0.770-0.996) | 0.044 |
| **CS tumor size (mm)** |  |  |  |  |  |  |
| ≤20 | Reference |  | Reference |  | Reference |  |
| 21-40 | 2.012 (1.721-2.351) | ＜0.001 | 0.989 (0.838-1.167) |  | 1.043 (0.883-1.232) | 0.619 |
| ≥41 | 3.496 (3.047-4.012) | ＜0.001 | 1.151 (0.982-1.350) |  | 1.120 (1.054-1.358) | 0.005 |
|  |  |  |  |  |  |  |
