# Supplementary material for: A nomogram incorporating treatment data for predicting overall survival in gastroenteropancreatic neuroendocrine tumors: a population-based cohort study
Source: Int J Surg. 2024 Jan 19;110(4):2178–86. doi: 10.1097/JS9.0000000000001080 (PMC11020034; doi:10.1097/JS9.0000000000001080)
Supplement: SUPPLEMENTARY MATERIAL [file js9-110-2178-s004.docx]

| **Items** | **Nomogram** | **AJCC system** | ***P*-value** |
| --- | --- | --- | --- |
| Training set |  |  |  |
| AIC | 20182.55 | 21208.11 |  |
| C-index (95% CI) | 0.816 (0.804-0.828) | 0.734 (0.720-0.748) | ＜0.001 |
| Testing set |  |  |  |
| AIC | 21501.94 | 22553.98 |  |
| C-index (95% CI) | 0.822 (0.812-0.832) | 0.743 (0.729-0.756) | ＜0.001 |
| AIC, Akaike Information Criterion; AJCC, American Joint Committee on Cancer. | | | |
